# Implementing alcohol screening and brief intervention in primary care: identifying barriers, proposing solutions

**DOI:** 10.1186/1940-0640-10-S1-A24

**Published:** 2015-02-20

**Authors:** J Aaron Johnson, J Paul Seale

**Affiliations:** 1 Institute of Public and Preventive Health, Georgia Regents University, Augusta, GA, 30912, USA; 2 Department of Family Medicine, Medical Center of Central Georgia and Mercer University School of Medicine, Macon, GA, 31206, USA

## Background

Alcohol screening and brief intervention (SBI) can reduce heavy and harmful alcohol use [1-5]. The strongest evidence for SBI effectiveness is in primary care settings where meta-analyses studies show 10–30 percent reductions in alcohol consumption at 12 months [4,5].

Over the past decade, there have been significant efforts to support the adoption and implementation of SBI in general health-care settings, including $305 million in SBI service and training grants from the Substance Abuse and Mental Health Services Administration (SAMHSA), and approximately $17 million per year from the National Institute on Alcohol Abuse and Alcoholism for more than 40 SBI studies. In addition, numerous individual state initiatives have focused on SBI dissemination. Nonetheless, a recent Centers for Disease Control and Prevention survey found alcohol SBI is rarely performed [6]. Only 1 in 6 adults reported they had talked with a health professional about their alcohol use in the past year [6]. The present study uses data from two SAMHSA-funded projects to identify barriers to widespread implementation of SBI and propose potential solutions.

## Methods

Data are from two separate and distinct sources. Data from specialists performing SBI in an emergency department (ED) setting with over 4800 patients over 12 months are used to estimate potential revenues generated.

Family medicine and internal medicine residents in four clinics were administered questionnaires before SBI training and one year later. These data are used in reporting residents’ confidence in performing SBI, level of importance placed on SBI, and residents’ individual drinking behavior.

## Results

Barriers to SBI implementation include low reimbursement rates and limited payers for SBI codes, restrictions on who can bill SBI codes, restrictions on same-day billing for mental health and primary care services, and minimum time requirements for billing that are much longer than time requirements for tobacco cessation. Additionally, existing Medicaid codes are not active in many states. Revenue estimates from the ED study found revenues generated would not sustain specialist-delivered SBI, with eight FTE specialists generating approximately $65,000 per year.

While SBI training for residents results in increases in knowledge and confidence, most studies show little change in delivery of brief interventions [7-9]. Results from resident questionnaires indicate residents are more comfortable addressing patients’ drug use than alcohol use. One possible contributing factor is residents’ own drinking behavior. Residents’ past-year binge drinking rate (49.7%) is 20 percentage points higher than among other adults age 25–35.

## Conclusions

Based on existing literature and results from these two projects, we recommend formal recognition and credentialing of health promotion specialists who would be able to bill SBI codes. Specialists should be cross-trained to provide other billable services to make the position more sustainable. Policy changes should be enacted to address the aforementioned billing issues. Absent policy changes, technology may present an attractive alternative. High rates of screening are possible when screening questions are integrated into electronic health records. Computer and web-based brief interventions have shown promising results in clinical trials and could be implemented as a first step in a stepped-care SBI model, resulting in significant cost savings.
